# Short Telomere Length as a Biomarker Risk of Lung Cancer Development Induced by High Radon Levels: A Pilot Study

**DOI:** 10.3390/ijerph15102152

**Published:** 2018-09-30

**Authors:** Narongchai Autsavapromporn, Pitchayaponne Klunklin, Chalat Threeratana, Wirote Tuntiwechapikul, Masahiro Hosoda, Shinji Tokonami

**Affiliations:** 1 Division of Radiation Oncology, Department of Radiology, Faculty of Medicine, Chiang Mai University, Chiang Mai 50200, Thailand; pitchayaponne.kl@cmu.ac.th; 2 Department of Biochemistry, Faculty of Medicine, Chiang Mai University, Chiang Mai 50200, Thailand; palmiisciencephoto@gmail.com (C.T.); wirote.t@cmu.ac.th (W.T.); 3 Graduate School of Health Science, Hirosaki University, Hirosaki, Aomori 036-8564, Japan; m_hosoda@hirosaki-u.ac.jp; 4 Institute of Radiation Emergency Medicine, Hirosaki University, Hirosaki, Aomori 036-8564, Japan; tokonami@hirosaki-u.ac.jp

**Keywords:** radon, lung cancer, high radon levels, telomere length, biomarker, climate change, burning season

## Abstract

Long-term exposure to radon has been determined to be the second leading cause of lung cancer after tobacco smoking. However, an in-depth study of this topic has not been explicitly carried out in Chiang Mai (Thailand). This paper presents the results of an indoor radon level measurement campaign in dwellings of Chiang Mai using total of 110 detectors (CR-39) during one year. The results show that the average radon levels varied from 35 to 219 Bq/m^3^, with an overall average of 57 Bq/m^3^. The finding also shows that the average value is higher than the global average value of 39 Bq/m^3^. In addition, to examine the cause of lung cancer development among people with risk of chronic exposure to radon during their lifetime, 35 non-smoker lung cancer patients and 33 healthy nonsmokers were analyzed for telomere length. As expected, telomere length was significantly shorter in lung cancer patients than in healthy nonsmokers. Among healthy nonsmokers, the telomere length was significantly shorter in a high radon group than in an unaffected low radon group. To the best of our knowledge, our research provides the first attempt in describing the shortened telomeres in areas with high levels of environmental radon that might be related to lung cancer development.

## 1. Introduction

Lung cancer is a major cause of cancer-related death worldwide [1]. In Chiang Mai, the capital of Upper Northern Thailand, lung cancer is the most common cause of cancer mortality and incidence in males and the third for females. The major reasons for the increasing incidence rate are tobacco smoking, indoor radon and air pollution. In addition, Chiang Mai generally faces air pollution caused by agricultural biomass burning and forest fires in March [2]. The city is surrounded by mountains, where the heat, smoke and dust can be accumulated in a confined environment. This crisis leads to health hazards such as chronic respiratory diseases and lung cancer that have greatly affected Chiang Mai residents.

Radon (^222^Rn) and its short-lived progeny represent a naturally occurring radioactive gas produced by the radioactive decay of uranium-238 (^238^U). It has a half-life of 3.82 days and decays with the emission of multiple high linear energy transfer (LET) α particles before it becomes stable lead-210 (^210^Pb). These particles are more capable of causing severe deoxyribonucleic acid (DNA) damage of high complexity with a direct hit on the DNA of cells of the respiratory epithelium cells, increasing the risk of cancer when compared to low-LET radiation such as X rays or γ rays [3]. Therefore, long-term exposure to radon and its progeny are the major contributors to the general populations’ exposure to high natural background radiation [4,5]. According to the World Health Organization (WHO), radon is the second leading cause of developing lung cancer after tobacco smoking and the most important risk to nonsmokers [6].

Radon contamination in the environment can occur through indoor and outdoor air as it is be generally emitted from ground, rock, soil, water and building materials. Accordingly, the inhalation of smoke and dust contaminated with radon during the fire winter burning season in Chiang Mai can cause lung cancer during a lifetime of exposure. Based on epidemiological studies, it is estimated that the induction period of lung cancer caused by radon exposure is between 5 and 25 years in humans [7]. Therefore, with the increasing occurrence of lung cancer, population that are long-term exposure to radon over a period of time should undergo health examinations in order to determine their risk of lung cancer, particularly the young adults population.

Telomeres are complex DNA-protein structures located at the end of chromosome. They play a key role in protecting linear chromosomes from end-to-end fusion, recombination, and degradation [8,9]. Thus, changes in the length of telomere can lead to telomere dysfunction, which in turn could cause lung cancer development [10,11]. Previous studies indicate that relatively shorter telomere length measured in peripheral white blood cells is associated with an increased risk of lung cancer and it may be used as a cancer biomarker [12,13]. Based on this observation, we have hypothesized that exposure to environmental radon pollutions may affect telomere shortening. Therefore, we assess telomere length changes in peripheral blood mononuclear cells (PMBCs) from lung cancer patients selected among in nonsmokers and healthy nonsmokers with special emphasis on the low-and high residential radon levels in the study area.

## 2. Materials and Methods

### 2.1. Study Area

**Figure 1:**
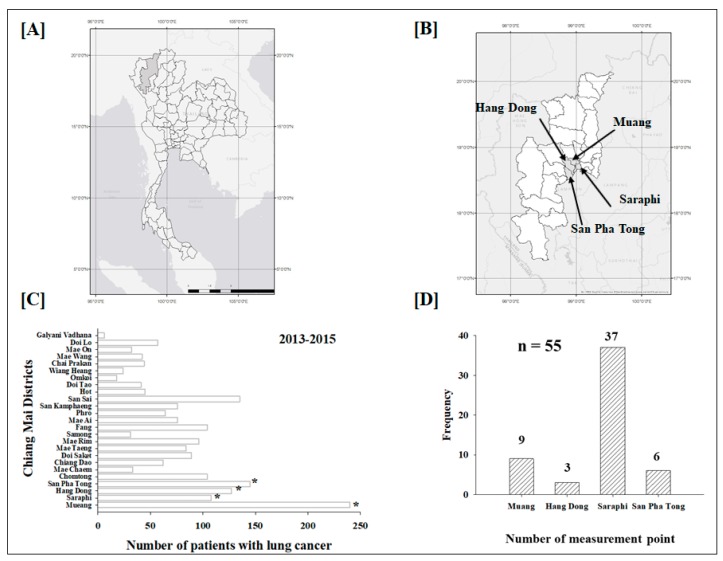
Geological map of the study area located in Upper Northern Thailand. (**A**) Geographical coordinates in Chiang Mai, Thailand. (**B**) Map of the study area. (**C**) Incidence of new lung cancer cases in the total of 25 districts of Chiang Mai from the years 2013 to 2015. (**D**) Frequency distribution of sampling sites in the study area.

**Figure 2:**
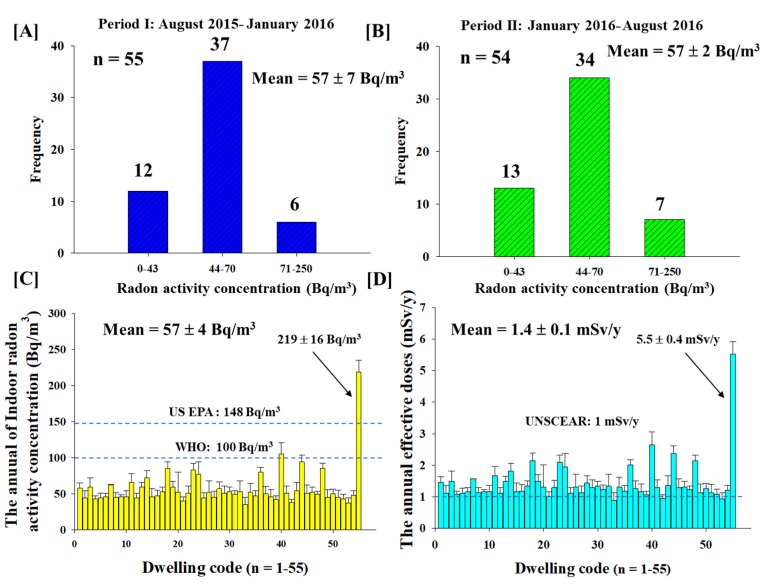
The radon activity concentrations and the effective doses of inhalation. (**A**) Distribution of the radon activity concentrations in Chiang Mai from August 2015 to January 2016. (**B**) Distribution of the radon activity concentrations in Chiang Mai from January to August 2016. (**C**) One-year average of radon activity concentrations in 55 Chiang Mai dwellings. (**D**) Annual mean effective dose for inhalation dose in 55 Chiang Mai dwellings.

Chiang Mai is located in the Upper Northern region of Thailand (Figure 1A). Figure 1B shows the study area in Chiang Mai, where a total of 110 radon detectors were placed in 55 houses (including lung cancer patients and healthy controls) in four different districts during the period from August 2015 through August 2016 (Figure 1D). The selected districts of Chiang Mai (including Muang, Hang Dong, Saraphi and San Pha Tong) were chosen with respect to the high number of new cases of lung cancer patients during 2013 to 2015 (Figure 1C). The classification of the radon activity concentration in the study area (Figure 1 and Figure 2) is separated into three groups: those with “low” (<44 Bq/m^3^), “moderate” (44–70 Bq/m^3^) and “high” (>70 Bq/m^3^) levels. 

### 2.2. Long Term Measurement of Indoor Radon Exposure Using a Passive Method

A passive type radon-thoron discriminative monitor called a RADUET using a solid-state track detector (CR-39) was used to measure the radon activity concentration. Each RADUET detector encompasses two diffusion chambers containing a CR-39 chip. They are deployed to record tracks of α particles and enable separate detection of radon (^222^Rn, half-life: 3.82 d) and thoron (^220^Rn, half-life: 55.4 s). The diffusion chambers have different ventilation properties which allow separate detection of radon and thoron. The performance of this RADUET detector was described by Tokonami et al. [14,15].

All of the RADUET detectors were placed at a height of one to two meters and 20 cm from the wall in the bedroom for all seasons. These detectors were placed in 55 dwellings for six months (Period I: before the burning season between August 2015 to January 2016). They were then replaced with new ones for another six months (Period II: during the burning season between January to August 2016). It should be noted that among the 55 RADUET detectors distributed during the fire winter burning season, 54 detectors were returned and were eligible for the measurement of radon concentration (Figure 2B). 

After the exposure, the detectors were sent to the Institute of Radiation Emergency Medicine, at Hirosaki University, Japan for analysis. Experimentally, the exposed CR-39 was chemically etched for 24 h in 6 M NaOH solution at 60 °C for clear visibility of α tracks before counting. Photos of α tracks were taken by a digital camera using an optical microscope. The number of α tracks were then counted using the image-J software. The average radon (C_RN_) -and thoron activity concentrations (C_Tn_) were calculated based on Equations (1) and (2) as follows [14,15,16]:

(1) $$\;C_{RN}=\;\frac{\rho }{kt}\;$$

(2) $$\;C_{TN}=\;\frac{\rho }{kt}\;$$

where *ρ* is α track densities (track/cm^2^) corrected by background track density. *k* is the respective conversion factor from α track densities to radon and thoron activity concentration [(tracks/cm^2^/h)/(Bq/m^3^)]. *t* is the exposure time (h). As a core of our study, the results obtained reveal that there is the relatively lower level of thoron than radon in all the dwellings from our study (data not shown).

### 2.3. Short Term Measurement of Indoor and Outdoor Radon Using an Active Method 

A pulse-type ionization chamber (AlphaGUARD) was used to measure the daily variation of indoor and outdoor radon exposure in the locations with highest radon concentration dwellings in Chiang Mai (Figure 2E). The observation was performed during the fire winter burning season between 13–16 March 2017. The AlphaGUARD was calibrated in the radon calibration chambers at the National Institute of Radiological Sciences (NIRS)-National Institutes for Quantum and Radiological Science and Technology (QST), Japan. The reference value of the radon concentration in the radon chamber was based on the measurement values obtained from the standard ionization chamber. The details of the radon calibration chamber and the calibration conditions of AlphaGUARD at NIRS-QST were previously described [17].

### 2.4. Estimation of the Average Annual Effective Dose 

The average annual effective dose caused by the inhalation of radon in 55 dwellings was calculated by the following Equation (3):

*E (mSv/y)* = *C* × *F* × *O* × *T* × *DCF* (3)

where *E* is the annual effective dose (mSv/y), *C* is the indoor radon activity concentration (Bq/m^3^), *F* is the equilibrium factor of radon using worldwide assumed value (0.4), *O* is the annual indoor occupancy factor (0.8), *T* is the time of exposure in a year (8760 h/y), and *DCF* is the inhalation dose conversion factor of 9 nSv/(Bq⋅h/m^3^) [17].

### 2.5. Soil Sample Preparation and Analysis

Soil samples were collected from the dwellings with the highest activity concentration of radon in the study area. The samples were air-dried and sieved through a 250 μm mesh size sediment sieve in order to homogenise them and remove large grains. The samples were then dried at a temperature of 110 °C to a constant weight. Then, the dried samples were pulverized into a fine powder and sieved through a 250 μm mesh size sediment sieve. Subsequently, the samples were packed into airtight plastic containers and sealed to prevent the escape of radon (^222^Rn) and thoron (^220^Rn). Prior measurement was conducted, the samples were stored for at least 30 days in order to establish secular equilibrium between ^226^Ra, ^232^Th and their radioactive progenies. All samples were analyzed for activity concentration of ^226^ Ra, ^232^Th and ^40^K using a typical high-resolution gamma spectroscopy as previously described [18].

### 2.6. Ethics Statement

Ethical clearance was obtained from the Research Ethics Committee in the Faculty of Medicine, Chiang Mai University, Thailand (Research ID: 2559-04001, 2559-04252). All participants were required to sign an informed consent in accordance with the Declaration of Helsinki before the enrollment.

### 2.7. Study Design and Characteristics of Study Population

The transitional study includes 35 lung cancer patients (17 males and 18 females) and 33 healthy nonsmokers (15 males and 18 females) in Chiang Mai with respect to age range, between 20–87 years and sex (Table 1). There are no genders, histological or stage restrictions in this study. We recruited patients who were newly diagnosed with lung cancer between 2014 and 2017 at Maharaj Nakon Chiang Mai University Hospital, in Chiang Mai, Thailand. It should be mentioned that all lung cancer patients used in this study were non-smokers or former smoker (patients having stopped smoking >15 years at the time of interview). All tumors were diagnosed as non-small cell lung carcinoma. Then, we randomly selected nonsmokers subjects that comprise 11 low residential radon (five males and six females) and 22 high residential radon (10 males and 12 females), who had lived in the study area of radon measurement during the past 5 years or more. In addition, healthy nonsmokers were individuals who had undergone minor, non-oncologic, ambulatory surgery and never smokers (less than 100 cigarettes smoked in lifetime or never as much as one cigarette per day during six months). All participants were interviewed by trained researchers using a questionnaire with predefined questions. They were asked about different aspects of their lifestyle, such as smoking history, environmental tobacco smoke exposure and working history. 

### 2.8. Blood Samples and Genomic DNA Extraction

Immediately after completing interview, blood samples (10 cc) were collected from participants in ethylenediaminetetraacetic acid (EDTA) tubes by trained phlebotomists. Then, PMBCs were collected from blood samples by gradient density centrifugation through HiSep (HIMEDIA, Mumbai, India) and stored at −80 °C before they were used. In our study, we used Blood DNA Extraction Kit (OMEGA, Norcross, GA, USA) to extracted genomic DNA from the PMBCs. 

### 2.9. Telomere Length Measurement 

The relative telomere length (T/S) from genomic DNA (20 ng/mL) was determined with a monochrome multiplex-quantitative real-time polymerase chain reaction (MMQPCR) method, as previously described [19,20]. Basically, the T/S value was calculated as the ratio of telomere repeat copies (T) to single-copy gene copy number and albumin gene (S) to represent the average length of telomere, which by definition is 1.00. In each sample, the quantity of telomere repeats and single copy gene copies were determined in comparison to those reference samples. All samples were assayed in triplicates in order to minimize the sample to sample variation. Once PCR has completed, the Applied Biosystems QuantStudio™ 6 Flex Real-Time PCR analysis software was used to determine the T and S values for each experimental sample based on the standard curve method (Applied Biosystems, Foster City, CA, USA). The results were expressed in terms of T/S ratio. The coefficient of variation in repeated measurements of the samples was 4.6%.

### 2.10. Statistical Analysis

All data presented in this paper were analyzed based on means ± SD. The characteristic of this study population were summarized using frequencies. The differences in telomere length between groups were assessed using the Wilcoxon rank-sum test. Linear regression analyses were performed to assess the relationship between age and telomere length; R^2^ values are given. All statistical analysis were performed by using the softwares Sigma Plot 10 (Systat Software, San Jose, CA, USA) and GraphPad Prism 7.05 (GraphPad Software, La Jolla, CA, USA); A *p* value of 0.05 or less between groups was considered to be significant. 

## 3. Result and Discussion

It has been recognized that the incidence of lung cancer in the Upper Northern region of Thailand, specifically in Chiang Mai Province (Figure 1A) is much higher than that of other cancer types and it is among the highest in Thailand [2]. The lung cancer incidence among Northern Thai women reaches the highest in Asia. However, the causes of this lung cancer incidence has not been clearly spelled out by any research work. There are evidences indicating that tobacco smoking, air pollution and indoor radon are associated with a high incidence of lung cancer. Previous studies reported by nationwide surveys of indoor radon concentrations during 1996 to 2000 show the range of radon from 1 to 1974 Bq/m^3^ with the activity concentrations of radon in Northern regions are among the highest in Thailand [2]. In fact, an increase in radon levels of 100 Bq/m^3^ is associated with approximately 16% increased risk of developing lung cancer [6,7]. Therefore, long-term measurement of indoor radon and its progeny in resident dwellings is very important from a health hazard point of view. Nevertheless, current research works dealing with the radon concentration measurement in Chiang Mai generally suffer from some limitations, including lack of long term measurement of radon levels, and absence of information about radon activity concentrations during the fire winter burning season.

To overcome some of above limitations, this paper describes the long-term measurement of indoor radon activity concentration using RADUET detectors (a total of 110 detectors) in 55 dwellings for a period of one year. The results given in Figure 2A show that the activity concentration of radon was ranged from 30 ± 19 Bq/m^3^ to 229 ± 44 Bq/m^3^ with a mean value of 57 ± 7 Bq/m^3^ within the period of August 2015 to January 2016. We found that out of 55 dwellings, 12 dwellings had less than 44 Bq/m^3^ of radon, 37 dwellings had above 44 Bq/m^3^ and six dwellings had high radon over 71 Bq/m^3^. As shown in Figure 2B, the radon activity concentration measured at 54 dwellings was obtained during the burning season from January to August 2016. The radon activity concentrations ranged from 34 ± 4 Bq/m^3^ to 209 ± 12 Bq/m^3^ with an overall average value of 57 ± 2 Bq/m^3^. As displayed in Figure 2C, 13 dwellings had radon levels below 43 Bq/m^3^, 34 dwellings had ones above 44 Bq/m^3^ and seven dwellings has high radon over 71 Bq/m^3^. Based on our study, the average of radon activity concentrations showed no significant difference (*p* = 0.76) between before (summer and rainy season) -and during the burning season (winter and summer season) in Chiang Mai province (Figure 2A,B). This indicates that there is no relationship between the indoor radon levels and seasonal variation caused by the influence of climate changes. Moreover, the annual average of radon activity concentrations (57 ± 4 Bq/m^3^) was found to be higher than the typical global average of 39 Bq/m^3^ [6], but it was still below the recommended action level of 100 and 148 Bq/m^3^ reported by the WHO and United States Environmental Protection Agency (US EPA), respectively. As a consequence, the measurements signify within safe limits, as shown in Figure 2C. In addition, both Figure 2A,B show that a maximum value of radon activity concentrations was obtained in the same dwelling locating around the San Pa Tong area. This is due to the fact that the high levels of radionuclides (^226^Ra, ^232^Th and ^40^K) in the soil, poor ventilation and lifestyle of the dwellers which caused an increase in the radon levels at the time the measurements were carried out [18]. Additionally, the geography of Chiang Mai can be considered as another important factor as the location is surrounded by mountainous terrain and the granite areas that may be a source of high deposit of radon levels and poor ventilation systems. The preliminary results of our study will be used to develop the radon contour map of the Chiang Mai Province in the future. 

Figure 2D shows the annual average effective dose caused by the inhalation of indoor radon for each dwelling. The annual average value of effective doses in the study area was 1.4 ± 0.1 mSv/y with the maximum value of 5.5 ± 0.4 mSv/y, which was slightly higher than the reference level for general public (1 mSv/y) as reported by the United Nations Scientific Committee on the Effects of Atomic Radiation (UNSCEAR). Although, the average value shown exceeds the worldwide average of 1.15 mSv/y [5]. It is still below the limit of the recommended action level of 3–10 mSv/y as reported by the International Commission on Radiological Protection (ICRP). Therefore, they are within the acceptable values from the radiation protection point of view [21]. Besides, based on the Linear No Threshold (LNT) model, the levels of low doses of high-LET α particles from radon that are considered safe are unknown at this time [22]. Therefore, low levels exposure of radon over a long period of time is still a potential factor accounting for lung cancer development. This indicates that effective actions must be taken to reduce the radon activity concentrations in the dwellings since the reduction of indoor radon exposure will reduce the incidence of lung cancers. According to the As Low as Reasonably Practicable (ALARA) concept in radiation protection, recommended strategies such as ceilings floor and/or wall, increasing the ventilation under the floor are worth to be considered [6].

**Figure 3:**
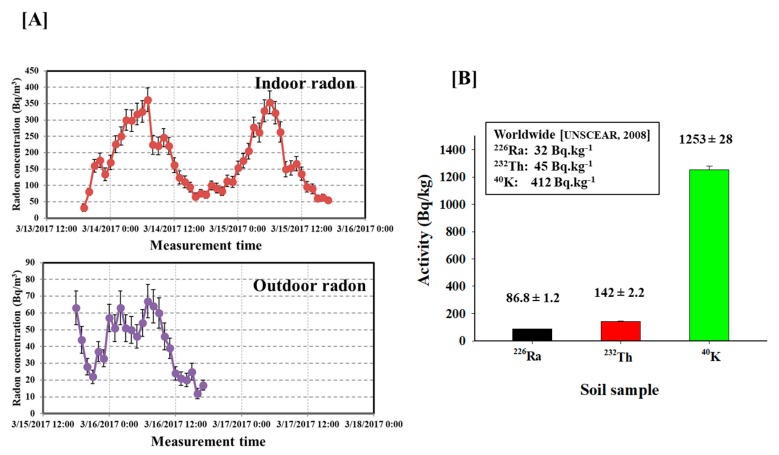
Measurement of radioactivity concentration in air and soil between 13–16 March 2017 in the dwelling with the highest concentration of radon during the fire winter burning season. (**A**) Temporal variation of radon activity concentrations measured using the AlphaGUARD. (**B**) Activity concentration of ^226^ Ra, ^232^Th and ^40^K in the studied soil samples.

To understand the levels of indoor and outdoor radon and the risk to the general public during the fire winter burning season in Chiang Mai using the AlphaGUARD, the actual quantification of radon concentration is needed. Figure 3A shows time variations of indoor and outdoor radon activity concentrations measured in the highest background radiation in study area. We found that within 3 days, the levels of indoor radon varied from 55 ± 9 Bq/m^3^ to 362 ± 36 Bq/m^3^ with the mean value of 176 ± 8 Bq/m^3^. For outdoor radon, the radon activity concentrations varied from 12 ± 3 Bq/m^3^ to 67 ± 10 Bq/m^3^ with an average value of 41 ± 2 Bq/m^3^. Clearly, the indoor radon activity concentration during burning season is relatively higher than the recommended action level reported by the WHO and US EPA [5,6]. Interestingly, the outdoor radon during the burning season shows an average value about four times higher than the world average of outdoor radon level [21]. Consequently, further fine-grained research with extensive experiments is essential to be conducted in order to elucidate the effects of radon during the winter burning season and normal condition on public health in Chiang Mai Province.

Furthermore, the activity concentration of the radionuclides, ^226^Ra, ^232^Th and ^40^K in the soil samples collected from dwelling with very high radon levels were measured using gamma spectral analysis as given in Figure 3B. The average activity concentration of naturally occurring ^226^Ra, ^232^Th and ^40^K were 86.8 ± 1.2, 142 ± 2.2 and 1253 ± 28, respectively. All values in the present study are higher than the worldwide values [5,23] and as expected, the soil gas radon concentration (^226^Ra) surrounding this dwelling naturally represents the main source of indoor radon activity concentration (Figure 3A,B). It may also cause by the geological and geographical such as a higher uranium contents in the underground soil. Hence, high levels of natural radioactivity in the soil from these areas may pose health risks for general public. Therefore, it is important to study the health effects from radon and terrestrial radionuclides on human population residing in this location. These observations are also in line with studies of soil samples from similar geological condition in Chiang Mai carried out earlier by Krisananuwat et al. [18].

With regard to the health effects of radon during the biomass burning season and human activities. Figure 3A highlights the radon activity concentration that was relatively higher during morning hours (between 6:00 a.m. and 7:00 a.m.) compared to late evenings (between 4:00 p.m. and 5:00 p.m.). Importantly, the difference in the values of indoor radon attributes to the metrological conditions such as temperature which appears to affect the radon levels. Notably, the daily variation of indoor radon levels depends on human behavior [17]. Human activities may enhance indoor radon levels as residents usually spend more time at home and workplace. Therefore, the knowledge of radon levels during the fire winter burning season is essential in assessing population exposure. More studies focused on the detailed analysis of these findings should be performed.

There is a growing evidence from epidemiological studies supporting a stronger association between higher levels of indoor radon exposure with lung cancer risk, and a very low radon activity concentration that may also can cause lung cancer [5,6,22,23,24,25]. This means that a relative increase for development of lung cancer in residential radon exposure can occur even at low exposure. It is to be noted that most of indoor radon-induced lung carcinogenesis are among those also exposed to cigarette smoke [24]. This situation causes us to determine a potential biomarker for early detection of lung cancer in high radon areas as well. To the best of our knowledge, this is the first study working on the examination of telomere length can be used as biomarker risk of lung cancer development induced by high levels of environmental radon exposure in Chiang Mai.

**Figure 4:**
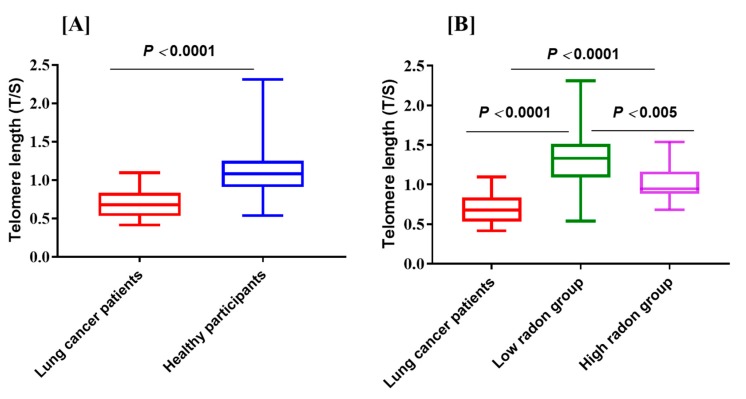
The relative telomere length (T/S value) in peripheral blood leukocytes in lung cancer patients and healthy controls with respect to low- and high radon areas. (**A**) Short telomere length in lung cancer patients. (**B**) Short telomere length in lung cancer patients as compared with healthy controls with respect to low -and high radon areas.

In the present study, leukocyte telomere length is basically determined from blood samples of 35 lung cancer patients in nonsmokers from Chiang Mai Province and 33 health nonsmokers from low and high level of radon of the study areas (Table 1). Importantly, the influence of smoking was not reflected in our study. As illustrated in Figure 4A, we observed a statistically significant decrease (*p* < 0.0001) in the mean telomer length (T/S ratio) between lung cancer patients and healthy individuals, where shortening telomere length was associated with an increased risk of lung cancer. These results are similar to other reports suggesting that shortening of the telomeres may be a risk factor of lung cancer [10,12,25,26]. We compared healthy nonsmokers (Figure 4B), that consists of 11 participants exposed to low radon levels and 22 participants as high radon group of the study area (Figure 1 and Figure 2). Interestingly, among the healthy nonsmokers, the telomere length was significantly (*p* < 0.005) shorter in high radon group compared to low radon group. Based on these finding, shorter telomere length can be considered as one of the development factors of indoor radon exposure-induced lung cancer. This notion was further supported by our findings that a total of 25 protein expression (such as nuclear protein localization protein 4, GTP-binding protein 6, interleukin-13 receptor subunit alpha-1) in high radon group and lung cancer patients were significantly up-regulated than low radon group (data not shown). Furthermore, it is important to note that the female had shorter telomere length than the male in all or subgroups of individuals (data not shown) and finding is similar to the report by Das et al. [27]. Collectively, the present results suggest that the presence of shortened telomere length in high radon exposure areas may be potential marker to lung cancer. This fact supports the concept that excessive telomere shortening may play a role in cancer development and progression [27,28,29]. One possible explanation for this finding is that telomere length shortening enables reduced proliferation and functions [27,28,29]. Shorter telomeres have also been reported in patients with lung cancer due to rapid cell proliferation and histological types of lung cancer [11,29,30]. Therefore, we need to take these factors (i.e., lung cancer risk and telomere length) into our study. Future investigation focused on this issue should be conducted for better understanding of the relationship between telomere length and lung cancer risk in high residential radon exposure.

**Figure 5:**
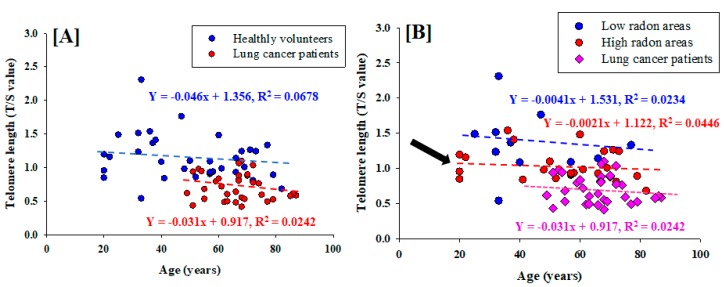
Correlation between age and relative telomere length (T/S value) in peripheral blood leukocytes in lung cancer patients and healthy controls. (**A**) Telomere length among lung cancer patients and healthy controls. (**B**) Telomere length among lung cancer patients and healthy controls with respect to low- and high level environmental radon areas.

In the last decade, telomere length had been pointed out as a useful for biomarker of biological aging and age-related cancer [13]. In order to further validate our findings, we have performed a linear regression analysis to study the relationship between the mean telomere length (relative T/S ratio) and age in lung cancer patients in nonsmokers and healthy nonsmokers. Our results in Figure 5A show a decrease in the relative T/S ratio in lung cancer patients with respect to age (R^2^ = 0.0242, y = −0.031x + 0.917). The results confirm that there is an age-dependent reduction of telomere length in patients with lung cancer. Similar trend was also observed in healthy nonsmokers (R^2^ = 0.0678, y = −0.0046x + 1.356), although the association was stronger in the healthy nonsmokers than lung cancer patients in nonsmokers. When healthy nonsmokers were categorized according to the levels of indoor radon in an individual’s dwelling (Figure 5B), telomere length decreased sharply with respect to age among those with high radon group (R^2^ = 0.0446, y = −0.0021x + 1.122) than low radon group (R^2^ = 0.0234, y = −0.004x + 1.531). An interesting finding in this present study is that shorter telomeres in younger individual (age range: 20–24 years) from high radon areas shows an increasing of developing lung cancer during lifetime exposure (Figure 5B, arrow symbol). This is based on the information that the youngest controls whose individual chronic exposure to radon is having increased oxidative stress on the bone marrow stem cells, causing telomere length shortening [31,32,33]. Therefore, short telomere has a correlation with lung cancer risk and leukemia in high residential radon exposure and the association is modulated by age and the cumulative lifetime exposure to radon [13,34,35]. Significantly, future larger studies are needed to entail the in-depth analysis of underlying mechanism of the age differences in the association between telomere length and lung cancer risk with respect to high level of natural radiation background areas.

The present study has several notable strength and limitations that may be summarized as follows. Firstly, we measured the radon activity concentration in individual’s dwellings during a period of one year. Therefore, daily variations of indoor radon activity concentrations due to the ventilation and season are eliminated. However, our finding suggests that there might be an environmental radon and air pollution interaction during the fire winter burning season in Chiang Mai. Secondly, this is the first study of using direct measurement on human, rather than country/area radon level to confirm the shorter telomeres are associated with high residential radon exposure. Thirdly, the study design has minimized the impact of cigarette smoking on telomere length in the residential radon exposure and lung cancer patients, where only non-smoking or former smoker were selected for this study. Finally, a limitation of our study is a small number of dwelling and individuals. The bias might influence the results of study areas such as the selection process, whereas, some factors can affect the length of telomeres such as age, gender, smoking status, length of exposure to radon, treatment and subtypes of cancer.

## 4. Conclusions

The results show that a high level of environmental radon pollution is a public health hazard concern in Chiang Mai Province. The association was modulated by climate condition, air pollution, human activities and the geographic locations are correlated with increased radon levels in a significant manner. Based on our finding, radon risk and appropriate actions for the reduction of radon should be efficiently communicated to residents. With regard to the relationship between the risk of developing lung cancer and telomere length, our findings suggested that short telomere lengths in high levels of environmental radon areas may be a biomarker risk inducing the development of lung cancer. For future works, we will pursue a more in-depth examination by performing the large-scale prospective cohort study for identifying potential biomarkers used of lung cancer risk in population in high radon areas as well as exploring their predictive value for clinical outcomes. Also, we will further investigate related-environmental radon exposure factors.

## Figures and Tables

**Table 1:** Characteristics of the study population.

Characteristics	Lung Cancer Patients (n = 35)	Healthy Nonsmokers (n = 33)		
		Low Radon (11)	High Radon (22)	Total (33)
**Age in years**, mean (SD)	66.4 (9.7)	43.5 (16.4)	53.6 (19.6)	50.2 (18.9)
**Gender**				
Male	17	5	10	15
Female	18	6	12	18
**Telomere length**, T/S ratio (SD)	0.7 (0.2)	1.35 (0.4)	1.0 (0.2)	1.12 (0.3)
